# Barriers and insights for maternal and newborn health service delivery redesign in Pakistan: a qualitative study

**DOI:** 10.1186/s12884-026-09459-6

**Published:** 2026-07-23

**Authors:** Tazeen Saeed Ali, Nousheen Akber Pradhan, Shehla Zaidi, Ammarah Ali, Wafa Zehra Jamal, Shabina Ariff

**Affiliations:** 1 School of Nursing and Midwifery, Department of Community Health Sciences, Aga Khan University, P. O. Box 3500, Stadium Road, Karachi, 74800 Pakistan; 2 Department of Community Health Sciences, Aga Khan University, Karachi, Pakistan; 3 University of Toronto, Toronto, Canada; 4 Department of Community Health Sciences, Aga Khan University, Karachi, Pakistan; 5 University College London, Gower Street, London, UK; 6 Department of Pediatrics and Child Health, Aga Khan University Hospital, Karachi, Pakistan

**Keywords:** Maternal and newborn health, Service delivery redesign, Community perspectives, Healthcare access, Respectful maternity care, Decision-making, Pakistan, Focus group discussions

## Abstract

**Background:**

Access to maternal and newborn health services in Khyber Pakhtunkhwa, Pakistan, is hindered by several barriers that impact the effectiveness of care. This study aimed to identify these barriers and gather community feedback on potential solutions for improving service delivery in the region through the implementation of Service Delivery Redesign.

**Methods:**

A descriptive-exploratory qualitative study was conducted using purposive sampling to select participants from three districts in Khyber Pakhtunkhwa: Kohat, Battagram, and Shangla. A total of 210 participants, including mothers, fathers, and Community Health Workers, took part in 18 Focus Group Discussions. Each discussion lasted approximately two hours. Thematic analysis was used to identify barriers to accessing maternal and newborn health services and to gather recommendations from the community on how to improve service delivery.

**Results:**

Key barriers to accessing maternal and newborn health services included limited availability of services, workforce shortages, disrespectful care, medicine shortages, and transportation challenges. Financial barriers, such as lack of knowledge about the Sehat Card and reliance on out-of-pocket payments, were also significant. Cultural factors, such as elder decision-making, patriarchal norms, and financial dependence, influenced healthcare-seeking behavior. Community stakeholders recommended improving infrastructure, supply chain management, respectful care, financial protection, and transportation.

**Conclusions:**

The study found several connected barriers that affect how mothers and newborns use healthcare in Khyber Pakhtunkhwa. People in the community pointed out problems such as a lack of medicines, equipment, and basic healthcare services. They also mentioned that there are not enough healthcare workers, which makes it harder to get good care. Many participants were also worried about being treated with respect and dignity. They said that how healthcare providers act and communicate has a big impact on whether people seek care and trust health facilities. Decisions about maternal healthcare were shaped by complicated family and social factors. Fathers, mothers-in-law, and money all played important roles in determining when and where women sought care. Including fathers in this study provided useful new insights into how they influence maternal health decisions, an aspect that has often been overlooked in earlier research in Pakistan. Even with these challenges, people in the community said they strongly prefer giving birth in health facilities. They listed several areas that need improvement, including more medicines and supplies, more staff, easier access, and care that is more respectful and patient-focused. These results show that there is a need for broad strategies that tackle both problems in the health system and the social issues that affect healthcare use. Redesigning services based on what the community says is needed could help ensure everyone has fair access to high-quality maternal and newborn care in Khyber Pakhtunkhwa. To overcome these barriers, it is important to strengthen primary healthcare centers, train healthcare workers to focus on patients, improve transportation, raise public awareness, and improve the Sehat Card program. These steps could help improve the health of mothers and newborns and lower preventable illness and death in Khyber Pakhtunkhwa.

## Introduction

It is critical to address inequities in maternal and neonatal health (MNH) in Low and middle-income countries [1]. The inadequate healthcare facilities in Pakistan’s rural areas make MNH service provision challenging. A few major challenges are a lack of community awareness, poorly run programs for community health workers, problems with governance that impact the operations of healthcare facilities, and insufficient transportation networks. Prenatal care, childhood immunizations, routine infant care, and facility-based births are the main areas affected by these obstacles. MCH service usage is hampered by several factors, including low knowledge of service benefits, ineffective public services, high private-sector prescription costs, transportation costs, a shortage of female healthcare workers, and women’s limited mobility because of security and cultural concerns [2].

For complex neonatal deliveries, acute neonatal conditions and antenatal care (ANC), communities prefer facility-based care provided by contracted institutions. These facilities operate under government agreements, receive funding and are monitored, and offer more standardized services. In contrast, non-contracted public health facilities function independently and lack formal government oversight. As a result, they are often perceived as unreliable, leading communities to rely on home-based deliveries, pharmacies, and alternative primary care providers. These are advanced primary care facilities that provide a wider range of services and can manage more complex conditions than standard primary care clinics. They have better resources and trained personnel, allowing them to deliver higher-quality and more comprehensive care.

In non-contracted areas, access to healthcare is limited due to facility-related challenges. As a result, communities often avoid facility-based services for routine infant care, normal deliveries, and postnatal care because of user fees, limited transportation options, and cultural norms [3].

Despite the introduction of cutting-edge MNH initiatives to improve outcomes, 810 women died each day due to avoidable complications in 2017. The severity of the problem is highlighted by the fact that 94% of these deaths occur in low- and middle-income countries (LMICs) [4]. The first 28 days of life are the most vulnerable for infants, accounting for nearly half (47%) of under-5 mortality worldwide, despite a drop in neonatal deaths from 5 million in 1990 to 2.4 million in 2019 [5]. A comprehensive strategy addressing community demand and health system supply, covering care from prenatal to postpartum, is required to achieve the Sustainable Development Goal (SDG) objectives (2015–2030) of 70 maternal deaths per 100,000 live births and 12 infant deaths per 1,000 live births [6, 7]. Pakistan has established a reputation as one of the riskiest countries for birthing because of its constant high Newborn Maternal Ratio (NMR) reports. The nation’s maternal mortality ratio (MMR), which was 186 in 2019 and remains high on the global MMR list, especially in rural areas, where it is 26% higher than in urban areas [8]. These difficulties result in less-than-ideal experiences of caregiving because of a variety of social determinants of health and aspects of the health system that impact expectant moms, new mothers, and infants [9]. Despite Pakistan’s commitment to the United Nations’ SDG progress in lowering maternal and newborn mortality is still slower than expected. Of all the births in 2019, almost 60% took place in hospitals; yet, local data indicates that MNH care is insufficient. These challenges include infrastructure deficits, limited access for expectant mothers, insensitive treatment, financial constraints, and cultural barriers that hinder facility-based deliveries [10, 11].

Maternal and Newborn Health (MNH) outcomes can be improved using a comprehensive strategy called “Service Delivery Redesign” (SDR) [12, 13]. It anticipates all deliveries occurring in well-equipped hospitals that can handle complications, with primary care centers offering excellent Antenatal Care (ANC) and Postnatal Care (PNC). In order to guarantee quality, enhance accessibility, and raise demand for high-quality MNH services, the SDR also entails intersectoral investments [14]. Before implementing this reform, it is important to explore the viability of SDR since the redesign process is complicated and stakeholder insights are essential for overcoming the obstacles to MNH care and for identifying potential solutions [15].

### Aim

To explore the perceptions, experiences, and care-seeking practices of community stakeholders—including pregnant women and mothers of young children, fathers, and CHWs —regarding maternal and newborn health services, and to identify community-perceived barriers, preferences, and conditions for implementing Service Delivery Redesign in Khyber Pakhtunkhwa, Pakistan.

### Objectives

1. To explore community care-seeking practices and decision-making dynamics related to maternal and newborn health services, including antenatal care, delivery, postnatal care, and newborn care.
2. To examine community experiences and perceptions of maternal and newborn health services, including quality of care, referral pathways, and interactions with public and private health facilities.
3. To identify key barriers and facilitators influencing access to maternal and newborn health services, including geographic access, transportation, financial costs, availability of providers, and sociocultural norms.
4. To assess community perceptions, acceptability, and enabling conditions for the implementation of Service Delivery Redesign (SDR) for maternal and newborn health care.

### Study design

This was an explorative qualitative study designed to deeply understand the cultural norms, beliefs, and systemic barriers that influence healthcare utilization for maternal and newborn health in the KP province. Focus group discussions (FGDs) were conducted with three key stakeholder groups mothers, fathers, and CHWs to gain insights into community health-seeking behaviors and to assess preferences for improving MNH service delivery through SDR. The study was designed to identify community-perceived barriers and challenges in accessing maternal and newborn health (MNH) services and to generate community-informed recommendations for strengthening health system responsiveness and optimizing service delivery to improve the accessibility and quality of MNH care.

### Study setting

This study was conducted in the Khyber Pakhtunkhwa (KP) province of Pakistan, which is located in the northwestern region of the country. KP is characterized by diverse geography, from mountainous terrains to more urbanized districts. The province shares an international border with Afghanistan. The province comprises 35 districts and includes a large proportion of mountainous and rural areas where access to healthcare services remains challenging. This study focused on three districts including Kohat, Battagram, and Shangla. These were selected due to their maternal and newborn health service delivery gaps and comparatively poor MNH outcomes. Among these, Kohat is relatively more urbanized, while Battagram and Shangla are predominantly rural districts characterized by mountainous terrain, dispersed populations, and limited access to healthcare facilities. These districts provided a representative context to explore the community’s views on MNH services and potential SDR implementation.

### Participants and characteristics

A total of 210 participants from the three districts participated in 18 Focus Group Discussions (FGDs). Participants were selected using purposive sampling to represent three key community groups:

### Community Health Workers (CHWs)

These frontline health providers deliver basic maternal and newborn health services at the community level, particularly in rural areas of the selected districts and play a key role in health education, referrals, and linking communities with health facilities.

#### Fathers of infants under one year of age

Fathers were included to explore their role in household decision-making and healthcare-seeking behaviors related to maternal and newborn health.

##### Pregnant women or recently delivered mothers (with infants under one year)

These participants provided firsthand insights into their experiences with antenatal, delivery, postnatal, and newborn care services.

The participants were selected from these groups to ensure a comprehensive understanding of the community’s health-seeking behaviors. In each district, six FGDs were conducted, with participants living both near and far from healthcare facilities.

Eligibility criteria for participants are provided in Table 1.

**Table 1:** Characteristics of focus group participants. Two additional participants were included in the Kohat fathers’ focus group discussions (one in the near group and one in the far group). The distribution of participants across districts is presented below

District	Mothers (*n*)	Fathers (*n*)	CHW/CHWs (*n*)	Total
Kohat	30	22	30	82
Battagram	22	21	21	64
Shangla	20	20	24	64
Total	72	63	75	210

Kohat: 30 mothers, 22 fathers, and 30 CHWs

- Battagram: 22 mothers, 21 fathers, and 21 CHWs
- Shangla: 20 mothers, 20 fathers, and 24 CHWs

Overall, the study included a total of 210 participants across three districts of Khyber Pakhtunkhwa, Pakistan, comprising 72 mothers or pregnant women, 63 fathers of infants, and 75 Community Health Workers. Table 2 showing the eligibility criteria for participants.

**Table 2:** Eligibility criteria for participants

1) Mothers ➢ Pregnant women ➢ Recent home or facility-based delivery within the last year with at least one live child ➢ Reproductive age: 18–49 years ➢ Includes both educated/uneducated and working/non-working mothers ➢ Delivery outcome must not include stillbirth, miscarriage, or infant death ➢ Excludes mothers-in-law, aunts, and healthcare providers
2) Fathers ➢ Father of infants under one year of age. ➢ Combination of group: Unemployed/employed ➢ Excludes: Uncles, father-in-law, and any healthcare provider.
3) CHWs ➢ Working at least 8–10 years in the selected communities ➢ Working in the same district with the nearest facility.

### Data collection process

Focus group discussions (FGDs) were conducted using semi-structured guides to explore health-seeking behaviors, barriers, and preferences related to maternal and newborn health (MNH) services. Discussions were held in local languages, lasted approximately two hours, and were audio-recorded with consent, alongside detailed field notes.

Data collection was conducted using multiple tools, including semi-structured Focus Group Discussion (FGD) guides, audio recordings, and detailed field notes documented by the research team during each discussion. These tools enabled comprehensive capture of participants’ experiences, perceptions, and recommendations regarding maternal and newborn health services. Observation notes were also used, where relevant, to document contextual information and non-verbal interactions during data collection.

Participants including mothers, fathers, and Community Health Workers (CHWs) were purposively recruited from communities located both near to and far from health facilities to capture variation in access and care-seeking behaviors.

Data collection was conducted by trained qualitative researchers from multidisciplinary backgrounds (medicine, psychology, sociology, and anthropology), with prior experience in community-based and health systems research. The process was supervised by an experienced team lead in Health Policy and Management. All facilitators received training on study protocols, ethics, informed consent, and FGD moderation.

Audio recordings were transcribed verbatim and translated into English. Written informed consent was obtained from all participants. The number of FGDs was guided by data saturation, with data collection continuing until no new themes emerged across diverse participant groups and settings.

## Sampling method

Purposive sampling was employed to ensure that participants with relevant experiences in the community were selected. Three key groups participated in the FGDs:

1. Fathers with infant under one year old
2. Mothers of reproductive age with at least one child under one year old
3. Community Health Workers (CHWs)

The FGDs were conducted in both urban and rural settings within each district, capturing a diverse range of experiences and barriers.

### Development of the FGD guide

The focus group discussion (FGD) guides were developed through a multi-step process. Initially, a comprehensive review of relevant literature on maternal and newborn health (MNH) service utilization, barriers to care, and service delivery redesign was conducted to identify key domains for exploration. Based on this review, preliminary FGD guides were drafted to capture community experiences, care-seeking behaviors, barriers to accessing MNH services, and perceptions regarding potential health system reforms.

The draft tools were subsequently reviewed by a panel of subject experts, including a gynecologist, pediatrician, social scientist, and a professor of health systems, to ensure content relevance, clarity, and appropriateness of the questions. Feedback from the expert review was incorporated to refine the questions and probes.

The revised tools were then tested through a dry run exercise to assess the clarity, flow, and cultural appropriateness of the questions. Following this, the guides were further reviewed during the training of data collectors, where each question and probe was discussed in detail. This development process helped ensure the content validity, clarity, and contextual relevance of the data collection tools before implementation. Minor contextual adjustments were made to ensure that the language and examples were appropriate for the local community settings (Table 3).

**Table 3:** Focus areas explored in focus group discussions by participant group

Participant Group	Focus Areas Explored in Focus Group Discussions
Mothers	- Challenges during pregnancy and childbirth.
	- Access to maternal healthcare services.
	- Cultural barriers to seeking healthcare.
Fathers	- Role in maternal and child healthcare decisions.
	- Financial and logistical challenges in accessing healthcare facilities.
	- Support for wives during childbirth.
CHWs	- Availability of healthcare resources in the community.
	- Experience and barriers faced in providing care.

### Data analysis

Thematic analysis was used to analyze the qualitative data from the FGDs. Key themes related to barriers to accessing maternal and newborn health services, healthcare-seeking behaviors, and community recommendations for service delivery redesign were identified. The analysis focused on identifying systemic issues and potential solutions based on community input, particularly those related to infrastructure, healthcare availability, financial protection, and cultural factors. Transcripts were reviewed repeatedly to achieve familiarity with the data. Four researchers independently coded the transcripts, and coding differences were discussed and resolved through consensus. Themes were developed, reviewed, and refined through iterative discussions among the research team.

### Trustworthiness of the study

To ensure rigor in the qualitative findings, strategies were applied to enhance credibility, transferability, dependability, and confirmability. Credibility was strengthened by conducting multiple focus group discussions across different communities, capturing diverse perspectives from mothers, fathers, and community health workers. Participant quotations were used to authentically represent experiences and support the emerging themes. Transferability was addressed by providing detailed descriptions of the study context, participants, and health system environment, allowing readers to assess the applicability of findings to similar settings. Dependability was ensured through a systematic research process using a structured FGD guide, recorded and transcribed discussions, and a transparent audit trail documenting each stage of data collection and analysis. Reflexive journaling was maintained to capture shifts in the researcher’s perspectives. Confirmability was achieved by grounding interpretations in participants’ narratives and documenting methodological and analytical decisions to ensure that findings were derived from the data rather than researcher assumptions. The audit trail included documentation of coding decisions, theme development, analytical discussions, and revisions made throughout the research process.

Additional measures were undertaken to strengthen the credibility and trustworthiness of the findings. All FGDs commenced with a pre-focus group discussion which enabled proper description of the study and area to facilitate internalization of the focus group responses. The use of triangulation, which combined data from FGDs and document analysis, was useful for verification of some of the findings and thus helped improve the credibility of the study. Moreover, participants were shown the summary of the FDS at the end of the discussions to make sure that their opinion had been reflected in the report. This process is commonly referred to as member checking. The process of data collection and data analysis and the results obtained have also been subjected to scrutiny and criticism using peer debriefing.

Additionally, the original intention of the study, the social phenomena studied, and the sample have been supplemented with additional information that will influence the transferability of the findings. The research also emphasized the detailing of the physical environment attributes of the research site which will assist readers in evaluating the level of generalization of the research outcomes.

## Results

Participant descriptive tables by district.

In Kohat district (Table 4), a total of 82 participants were included, comprising 30 mothers, 22 fathers, and 30 CHWs. The age of participating mothers ranged from 18 to 55 years, with parity ranging from zero to eight children. Most mothers reported being housewives, although a few were engaged in home-based work or teaching. Fathers represented diverse occupational backgrounds, including police personnel, drivers, farmers, forest guards, shopkeepers, laborers, army personnel, and unemployed individuals. The CHW participants included Community Health Workers and a Lady Health Supervisor serving multiple villages in the district.

**Table 4:** Participant characteristics – Kohat district

Participant Group	Number of Participants (*n*)	Age Range (years)	Number of Children (Range)	Occupation / Professional Role
Pregnant Women / Mothers	30	18–55	0–8	Mostly housewives; a few engaged in home-based work or teaching
Fathers of Infants	22	21–58	1–10	Police personnel, drivers, farmers, forest guards, army personnel, shopkeepers, labourers, unemployed
CHWs	30	~ 25–50	N/A	Community Health Workers and one Lady Health Supervisor serving villages in Billi tang Union Council
Total	82	—	—	—

In Battagram district (Table 5), 64 participants took part in the discussions, including 22 mothers, 21 fathers, and 21 CHWs. The age of mothers ranged from 19 to 39 years, with the number of children ranging from one to seven. Most women reported being housewives, though some were engaged in small home-based income-generating activities. Fathers were involved in occupations such as government service, farming, private sector employment, police service, and daily wage labor. CHW participants were frontline community health workers providing maternal and newborn health services within their respective catchment areas.

**Table 5:** Participant characteristics – Battagram district

Participant Group	Number of Participants (*n*)	Age Range (years)	Number of Children (Range)	Occupation / Professional Role
Pregnant Women / Mothers	22	19–39	1–7	Majority housewives; some involved in small home-based income activities
Fathers of Infants	21	21–45	1–8	Government employees, farmers, private workers, police personnel, daily wage earners
CHWs	21	~ 25–50	N/A	Community health workers providing MNH services in multiple villages
Total	64	—	—	—

In Shangla district (Table 6), 64 participants were included, comprising 20 mothers, 20 fathers, and 24 CHWs. Participating mothers were aged 20 to 36 years, with one to five children, and all reported being housewives. Fathers were engaged in occupations including taxi driving, shopkeeping, daily wage labor, forestry-related employment, private teaching, and unemployment. The CHW participants included community health workers and Lady Health Supervisors responsible for maternal and child health services across several villages and union councils.

**Table 6:** Participant characteristics – Shangla district

Participant Group	Number of Participants (*n*)	Age Range (years)	Number of Children (Range)	Occupation / Professional Role
Pregnant Women / Mothers	20	20–36	1–5	All participants housewives
Fathers of Infants	20	19–35	1–6	Taxi drivers, shopkeepers, daily wage labourers, teachers, forestry employees, unemployed
Community Health Workers (CHWs)	24	~ 25–50	N/A	CHWs and Lady Health Supervisors providing community maternal and newborn health services
Total	64	—	—	—

### Theme 1: gaps in comprehensive service delivery and medical supplies shortages

Participants reported substantial gaps in the availability and delivery of maternal and newborn health services across Kohat, Shangla, and Battagram. Widespread concerns were expressed regarding fragmented service provision and limited continuity of care across different levels of the health system. According to the participants, widespread dissatisfaction was voiced regarding the patchy and fragmented manner of MNH services that had no continuity between the facilities at the primary, secondary, and tertiary levels of healthcare. This lack of connectivity often resulted in patients going to multiple facilities, making access to health services both confusing and frustrating for the family.

Mothers in Kohat were first taken to Basic Health Units for antenatal care. Again, the shortage of facilities and infrastructure led to them being referred to tertiary government hospitals very frequently, which was both logistically and financially burdensome. A mother reported:

*“For the ANC visit, I go to BHU, but again, the facilities are limited here, so I have to visit tertiary care government hospital”.*

This was also the case in Shangla, where interviewees reported that the health providers referred the patients elsewhere because the local clinics had no diagnostic tests.

*“The doctor referred me to another doctor in the hospital because they did not have test facilities”.*

They reported that they rely on teaching hospitals to avail simple essential services under MNH in Battagram. In fact these respondents reported acute deficiencies in basic equipment; local facilities often did not have hospital incubators available. Therefore, families failed to access care for a considerable period even during cases of emergency as it emerged from the following interview. “When our newborn babies are sick after delivery, there are no incubators present in our ABC hospital (government hospital), so then children are referred to Abbottabad hospitals.”

These testimonies point to an important place where better coordination and resource management are required for mothers to deliver safely in those communities with care at an appropriate time.

Medicine and supply shortages further complicate these challenges in the delivery of the services. The MNH supplies have been found deficient in public healthcare facilities. Proper outfitting of government buildings to provide basic MNH products was called for by most community stakeholders, but certain medications and equipment were constantly missing or out of stock in the healthcare facilities. External purchase of medications and equipment meant higher costs for patients as well as more dissatisfaction within the community with public health facilities.

Medicine and supply shortages emerged as critical issues affecting the quality and accessibility of public healthcare services. Community members and healthcare providers expressed frustration over the limited availability of essential medications and functional equipment in hospitals, which often forced patients to bear additional expenses by purchasing supplies externally.

In Battagram, one mother highlighted the financial and logistical burden this creates for families when seeking care for their newborns:

*“When newborn babies get sick, they are admitted in DHQ hospital, but medicines are to be bought from outside as no medicines are available at the hospital”.*

Healthcare workers also detailed frequent stock shortages that limit patient care. In Shangla, a CHW noted that only a minimal supply of basic medications such as painkillers, misoprostol, folic acid, and vitamins is available. However, these supplies often run out by the beginning of each month, leaving patients with no option but to seek consultations without treatment:

*“There are mostly pain killers, misoprostol and folic acid or vitamin tablets available, but in very small quantities. Every month on the 5th date, the medicine stock-out occurs, and then only consultations are done”.*

The lack of functional equipment further impacts the quality of healthcare services. Essential machines, like ultrasound equipment and laboratory facilities, are often broken or unavailable, limiting diagnostic capabilities. Another CHW in Shangla described the difficulties this poses:

*“The equipment in the hospital has become nonfunctional, but they will tell you that we don’t have facilities”.*

### Themes 2: workforce and accessibility barriers

Transportation challenges and manpower shortages in the public healthcare facilities have caused hindrances in accessing MCH services across the three districts. Lack of available means of transport was obstacle to accessing medical care. The respondents claimed that most of the time, they had to arrange their own transport to get to the healthcare institutions either by hiring jeeps, private cars, or Datsun. This cost prevented many residents in the community from obtaining treatment. Eventually, there was a realization that there needed to be residential delivery. Many women failed to reach hospitals on time even after transport arrangements had been made.

*“In most emergency cases, we arrange vehicles at our own expense. It’s difficult for our people to arrange transport as no public transport is available, and the roads are also not in good condition” (Mother- Shangla).*

*“No ambulance service is available in our union council” (Father- Shangla).*

*“Transport is the biggest issue which stops us because the health facilities are far away, and transport is too costly, and it is hard to find public transport other than 7 to 8 am and after 5 pm, there is no private transport” (Father- Kohat).*

*“We arrange private car or Datson for patients which cost us around 2500 to 3000” (Father- Battagram).*

As it has been the tradition in places like Shangla districts or Battagram that they planned beforehand to ensure an ambulance reaches their region to pick up their pregnant women headed to RHC or DHQ. The distance as it is shorter in Kohat and medical facilities, making less travel time compared with Shangla and Battagram, therefore much more hazardous terrain makes travelling worse there.

Apart from the inaccessibility of transportation means, another challenge in accessing MNB care is the lack of personnel required to offer health services at public sector healthcare. During their focus group discussions, members pointed out that there would be frequent lack of doctors that forced members to seek services at a private healthcare facility.

*“Female doctor in XYZ hospital (government hospital) was doing a clinic. We waited 2 hours for her at the government hospital; then we visited her in the clinic” (Mother- Kohat).*

*“Government hospitals lack adequate facilities, and they always have no doctor whenever we go in an emergency” (Husband- Shangla).*

Participants perceived that the current role of Lady Health Workers was heavily focused on polio-related activities, with comparatively limited engagement in broader maternal and newborn health services. Several respondents reported infrequent community visits related to maternal and child health and expressed concerns regarding gaps in health education, follow-up, and community outreach. These perceptions suggest a need to strengthen the involvement of frontline health workers in maternal and newborn health activities at the community level.

*“There is no one who visits in the community for mother and child health; they only come to give polio drops to our kids; that’s they do regularly, but no other visit, no sessions or awareness on health or anything else” (Father- Shangla).*

*“We have never seen an CHW visit our home for mother and child health. They all take only their salaries and never visit the community” (Father- Shangla).*

### Theme 3: barriers to dignified and accessible care

The challenges cut across both quality and financial accessibility of maternal and neonatal care in public facilities. The participants repeatedly raised concerns about disrespectful treatment from healthcare providers and out-of-pocket costs that were unaffordable, which altogether barred equal access to healthcare, affected patient dignity, and destroyed trust in the public health system.

Communities reported cases of discourteous, negligent, and even derogatory attitudes from health workers towards mothers, with many reports of patient privacy violations. Women in labor, already in vulnerable situations, were reportedly treated without sensitivity and, at times, hostility. Health care providers were also said to favor patients on the basis of informal payments that created experiences of inequitable treatment among patients. Bribes are called “sweets money” and were said to also be offered following a birth, thus adding more financial burden on the emotional pressures of the family.

As a mother from Kohat stated, *“The conditions in government hospitals are worse. A woman is already in labor pain*,* but the nurses and dai (Traditional Birth Attendant) treat her harshly…they are butchers.”* Similarly, a father from Battagram shared his frustration with the lack of respectful treatment, expressing his hope that *“doctors would treat us fairly with respect without discrimination.”*

The quality of care, coupled with financial barriers, has been one of the most significant bottlenecks for families seeking access to services. Despite the implementation of the Sehat Card by the KP government to increase access to healthcare at low costs, there remain deficiencies in utilization. The poor often lack information regarding the benefits of the Sehat Card or are misguided, and some providers are said to charge extra payments to those not well-informed about their entitlements. Respondents said they were still left with most of the expenses even on Sehat Card because apart from drugs and transport costs were the highest most of the time. Therefore, if the family or patient could not afford all facilities, they opted to be delivered at home hence increased the cost of this birth attendant from savings loans or both.

A mother from Battagram detailed her experience, saying, *“We got an appointment in advance at the private hospital and faced no issue*,* but they charged from the Sehat Card*,* and we had to pay an additional 7*,*000 PKR for other services.”* Another father added, *“We also pay nurses after delivery; they ask us for sweets money.”*

These combined challenges of discriminatory and unequal treatment and lack of access to finance call for a better need for community education on Sehat Card schemes and more accountable measures to ensure equitable and accessible health care to all.

### Theme 4: decision-making dynamics

The community’s pervasive patriarchy and the attitudes of the elderly had a big impact on the delivery location choice. The household’s financial resources also played a major role. The growing influence of fathers in decision-making was noted as an emerging practice throughout the three districts, even if mothers-in-law continued to play a significant role in determining where pregnant women gave birth. The father has the option to choose the location of the delivery if he is self-sufficient financially. If not, the mother-in-law gets to make the call. A few responses mentioned that the couple decided on the delivery location together, which was a relatively unusual procedure.

*“We (Father) take decisions. If someone is earning money*,* then they can decide. If the husband is not earning*,* then Father suggests*,* and we should obey him” (Father- Shangla)*.

*“My mother decides because she knows when we will need to go to the hospital in case of emergency” (Father- Shangla)*.

Decision-making authority made by father for the choice of place of delivery is more commonly seen in Kohat as compared to the other two districts. Men, the head of the household, were reported to decide on hospitals as delivery places.

### Theme 5: community stakeholder preferences for service delivery redesign

Stakeholders identified multiple priority areas for improvement to strengthen facility-based deliveries and maternal and new born health service utilization in Khyber Pakhtunkhwa. Recommendations included improvements in health facility infrastructure, availability of medicines and equipment, staffing, transportation services, and the provision of respectful and patient-centered care. This covers updates to hospital architecture, the provision of comfortable beds, and the upkeep of necessary supplies, medications, and equipment in government hospitals. In addition to free services, the availability of oxygen supply, blood banks, and full-time medical staff, especially female doctors during the evenings, as well as increased visits by CHWs to community places, etc.

All three groups of respondents strongly addressed the transportation barriers poor households face by proposing that the government provide transportation and ambulance services. Some respondents also highlighted the 24/7 availability of services and facilities closer to their residences.

*“Government hospitals should be free all tests x-ray and other facilities should be available in the hospital 24/7” (Father- Battagram)*.

In light of their experiences with staff members who have been impolite and disrespectful, the mothers and fathers in the community suggested providing moms at the institutions with loving care. In the context of crucial support areas for boosting SDR, community awareness-raising and the involvement of men and elders especially fathers and pregnant women were regarded as critical factors. Across the three stakeholder categories, the identified SDR preferences stayed consistent. Refresher courses and training in MNH were pointed out by a few CHWs.

“*Since we were hired*,* no training has been provided to us up till now; if any training had been provided to us*,* we would have worked in our community and facilitated our community*,* particularly for ANC and PNC; this would have ultimately reduced the burden on government health facilities”. (CHW- Kohat)*

## Discussion

The results of the feasibility evaluation highlighted the urgent need for health system reforms to better address the unmet needs in maternal and neonatal health (MNH) care. Community stakeholders identified several key barriers, including the lack of a continuum of care, shortages in human resources, insufficient transportation, and demeaning behaviour from healthcare personnel, particularly in public hospitals. They also pointed to the scarcity of medical supplies and equipment, as well as the out-of-pocket costs women still face despite financial protection programs. In order to ensure women can easily access obstetric and neonatal care, these obstacles must be addressed through structural changes in the health system. The MNH redesign proposals discussed by the community called for significant improvements in several areas, including ensuring the availability of services, supplies, and equipment, and tackling financial barriers to care. One of the most cost-effective strategies to reduce preventable maternal and infant morbidity and mortality is to strengthen the maternal continuum of care, which encompasses antenatal care (ANC) during pregnancy, skilled birth attendance during delivery, and postnatal care (PNC) [16, 17]. Women who face difficulties accessing healthcare are more likely to experience gaps in this continuum, increasing their risk of maternal and neonatal complications or death [18].

### Comprehensive maternal and newborn healthcare services

Our study found that the continuum of care for mothers and newborns needs improvement due to the lack of comprehensive antenatal, delivery, and postpartum services at healthcare facilities closer to the community. As a result, mothers are often forced to travel longer distances to access these services at other facilities. The challenging physical terrain and limited transportation options further complicate access to distant healthcare centers. Similar findings were reported in an Indian study, where women were bypassing nearby facilities despite them being functional and traveling great distances to others. This not only increases the financial burden on families but also leads to inefficient use of the health system’s resources [17].

Research indicates that a less comprehensive continuum of care is associated with longer travel times to access medical services [19, 20]. The local study also found that while the health worker program neglected some maternal and child health (MCH) services, clients in areas with Lady Health Workers (CHWs) were more informed about antenatal care (ANC) and family planning [2]. Additionally, there was significant demand from the community for access to medical facilities, particularly for pregnancy and paediatric emergencies. However, this would require critical support, such as improved transportation networks and better-equipped medical facilities.

In western Kenya, Kakamega County became the first to implement the MNH Service Delivery Redesign project. While the feasibility assessment identified challenges such as transportation issues and a shortage of healthcare workers, it also revealed that the infrastructure could be redesigned to address these problems, with strong stakeholder support [15].

### Ensuring dignity and respect

Every woman deserves to be treated with dignity and respect, regardless of her race, ethnicity, age, religion, or financial status. Unfortunately, communities reported experiencing disrespectful and discriminatory behaviour from healthcare staff, particularly in public hospitals, alongside other shortcomings in the government’s ability to provide adequate MNH care. The disheartening attitudes of healthcare workers and the absence of effective supervision in public hospitals may be contributing to this mistreatment and misconduct. As a result, many community members avoid seeking facility-based services for well-baby care, routine deliveries, and postnatal care. This reluctance is further explained by other factors identified in the analysis, including user fees, long travel distances with limited transportation options, and cultural norms [3].

Our study’s findings are consistent with those from other health facilities in low- and middle-income countries (LMICs), where maternity care has been marked by prejudice, inadequate psychosocial support, and a condescending attitude toward women. These factors are associated with a decreased likelihood of women choosing to deliver at the same facility in the future [21–23]. A Kenyan study also highlighted concerns about being reprimanded by healthcare workers and denied care, with participants expressing widespread fear due to the negative attitudes of health staff. This fear affected not only those who had bad experiences but also influenced the decisions of other women and their social networks [24]. To ensure respectful, patient-centered care, it is essential to implement policies, provide training, and invest in improving the healthcare environment before rolling out MNH redesign initiatives. This will help build user confidence in the health system.

### Addressing funding gap

The study identified a lack of funding as a key limitation. Consequently, enhancing financial access through intersectoral investments is a fundamental principle of health system redesign for MNH care. Research shows that providing financial protection through insurance schemes or cash transfer program improves MNH outcomes by increasing the utilization of healthcare services [25–28]. Countries like Ghana, Nepal, Sierra Leone, and Zambia have introduced policies aimed at reducing financial barriers to healthcare access. Similarly, our study found that the Sehat Sahulat Program, implemented by the Khyber Pakhtunkhwa (KP) government, improved financial access for low-income individuals, aligning with findings from other studies [16, 17].

However, the program could have been utilized more effectively, as community members reported still paying some medical expenses out of pocket, which undermines the fundamental goal of providing a social safety net. Despite high awareness of the Sehat Card, a third-party study conducted in Khyber Pakhtunkhwa found that additional information about its various functional components was necessary. Furthermore, the review highlighted challenges in accessing distant hospitals and receiving healthcare from remote districts. The patriarchal system in Pakistan and other Southeast Asian countries continues to influence decision-making among men, which hinders women’s empowerment and can result in complications for women giving birth at home. Conversely, research indicates that women who are empowered to make decisions are more likely to utilize the continuum of care and choose facility-based deliveries [16, 20]. While traditionally, household heads such as parents or in-laws—have been the primary decision-makers, there is a noticeable shift towards couples making joint decisions about facility deliveries, with an increasing influence of husbands in this process, which is a positive development.

## Conclusion

The findings highlight systemic and structural barriers that limit women’s use of facility-based maternal and newborn care in the districts of Khyber Pakhtunkhwa. Although maternal and child health is a national priority, utilization remains constrained by poor transportation, difficult road access, long distances to facilities, and the financial burden of seeking care. Participants also reported concerns about the quality of services in public facilities, including shortages of skilled staff, medicines, and equipment, as well as negative provider attitudes that weaken community trust in the health system.

Despite these barriers, communities expressed a strong preference for hospital-based deliveries because of the perceived availability of skilled providers and emergency care. These findings underscore the need for coordinated health system strengthening that improves geographic access, facility readiness, and respectful maternity care. Strengthening these areas can support the implementation of Service Delivery Redesign strategies aimed at improving equitable access to safe maternal and newborn care in Khyber Pakhtunkhwa.

### Recommendations

To improve maternal and newborn health (MNH) services within districts of KP, several strategic actions are recommended. Primary healthcare (PHC) facilities and hospitals must be equipped with essential obstetric and newborn care resources, while healthcare staff should receive training to promote respectful, patient-centered care. Collaboration with the Ministry of Transport is crucial to improve road infrastructure and establish transport services closer to communities. Communication campaigns targeting family members, including elders and fathers, should be implemented to encourage facility-based births. Additionally, enhancing the Sehat Sahulat Program to reduce out-of-pocket costs will improve financial access to MNH services. Lastly, provincial health departments should evaluate district teams’ capabilities to address these gaps effectively, providing necessary support for these reforms.

### Strengths and limitations

This study offers valuable insights into community perspectives on maternal and newborn health service delivery by incorporating the views of multiple stakeholder groups, including mothers, fathers, and frontline community health workers across three districts of Khyber Pakhtunkhwa. The inclusion of fathers added an important dimension to the analysis, as their role in household decision-making and healthcare utilization is often overlooked in maternal and newborn health research. Gathering perspectives from different participant groups enabled comparison of experiences and strengthened the overall interpretation of the findings.

A further strength of the study was the inclusion of participants residing both near to and far from healthcare facilities, allowing exploration of barriers and healthcare experiences across varying geographic and service-access contexts. Conducting focus group discussions across diverse settings generated rich qualitative data and enhanced the contextual relevance of the findings. The use of systematic qualitative procedures, including verbatim transcription, independent coding by multiple researchers, consensus-based theme development, and maintenance of an audit trail, further strengthened the trustworthiness of the analysis.

Several limitations should be acknowledged. As a qualitative feasibility study, the findings provide in-depth insights into community experiences and perceptions but are not intended to be statistically generalizable to the wider population. Participants were recruited from only three districts of Khyber Pakhtunkhwa; therefore, the findings may not fully reflect experiences in other geographical or cultural settings. In addition, responses obtained during focus group discussions may have been influenced by recall bias or social desirability bias. Finally, this component of the research focused solely on community stakeholders and did not include perspectives from policymakers, managers, or other health system actors. Consequently, the findings should be interpreted alongside evidence generated from other components of the broader study.

## Data Availability

The datasets generated and/or analyzed during the current study are available from the corresponding author on reasonable request.

## Abbreviations

MNH: Maternal and Newborn Health
ANC: Antenatal Care
PNC: Postnatal Care
CHWs: Community Health Workers
KP: Khyber Pakhtunkhwa
